# {2-[6-(1*H*-Benzimidazol-2-yl-κ*N* ^3^)-2-pyridyl-κ*N*]benzimidazolato-κ*N*}(dicyanamido-κ*N*)(methanol-κ*O*)copper(II)

**DOI:** 10.1107/S1600536810050178

**Published:** 2010-12-11

**Authors:** Jingchun Hu, Jinfang Zhang, Weiming Zhang, Chi Zhang

**Affiliations:** a Molecular Materials Research Center, Scientific Research Academy, School of Chemistry and Chemical Engineering, Jiangsu University, Zhenjiang 212013, People’s Republic of China

## Abstract

In the title compound, [Cu(C_19_H_12_N_5_)(C_2_N_3_)(CH_3_OH)], the Cu^II^ atom is coordinated by three N atoms from an anionic 2,6-bis­(1*H*-benzimidazol-2-yl)pyridine (bbp) ligand, an O atom from a methanol mol­ecule and one N atom from a dicyanamide anion. The crystal structure is stabilized by O—H⋯N and N—H⋯N hydrogen bonds, forming a three-dimensional network.

## Related literature

For potential applications of benzimidazole derivatives and their metal complexes, see: Khaled (2003 ▶); Hay *et al.* (1998 ▶); Petoud *et al.* (1997 ▶); Liu *et al.* (2005 ▶); Boinnard *et al.* (1990 ▶); Mo *et al.* (2009 ▶); Addison & Burke (1981 ▶). For examples of other bbp-containing complexes, see: Wang *et al.* (1994 ▶); Bernardinelli *et al.* (1990 ▶).
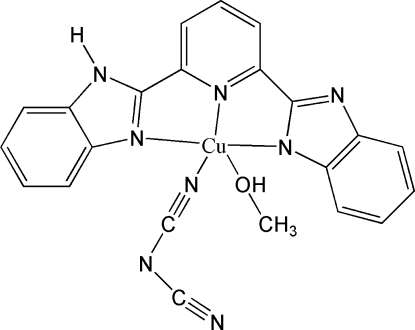


## Experimental

### 

#### Crystal data

[Cu(C_19_H_12_N_5_)(C_2_N_3_)(CH_4_O)]
*M* *_r_* = 471.98
Triclinic, 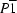

*a* = 6.8262 (14) Å
*b* = 12.189 (2) Å
*c* = 12.609 (3) Å
α = 101.74 (3)°
β = 99.03 (3)°
γ = 97.12 (3)°
*V* = 1001.2 (4) Å^3^
*Z* = 2
Mo *K*α radiation
μ = 1.13 mm^−1^
*T* = 293 K
0.20 × 0.16 × 0.12 mm

#### Data collection

Rigaku Saturn724 diffractometer
Absorption correction: multi-scan (*CrystalClear*; Rigaku, 2007 ▶) *T*_min_ = 0.806, *T*_max_ = 0.874
7787 measured reflections
3591 independent reflections
3294 reflections with *I* > 2σ(*I*)
*R*_int_ = 0.020

#### Refinement

*R*[*F* ^2^ > 2σ(*F* ^2^)] = 0.030
*wR*(*F* ^2^) = 0.072
*S* = 1.02
3591 reflections
290 parameters
H-atom parameters constrained
Δρ_max_ = 0.52 e Å^−3^
Δρ_min_ = −0.28 e Å^−3^

### 

Data collection: *CrystalClear* (Rigaku, 2007 ▶); cell refinement: *CrystalClear*; data reduction: *CrystalClear*; program(s) used to solve structure: *SHELXS97* (Sheldrick, 2008 ▶); program(s) used to refine structure: *SHELXL97* (Sheldrick, 2008 ▶); molecular graphics: *SHELXTL* (Sheldrick, 2008 ▶); software used to prepare material for publication: *SHELXTL*.

## Supplementary Material

Crystal structure: contains datablocks I, global. DOI: 10.1107/S1600536810050178/bt5405sup1.cif

Structure factors: contains datablocks I. DOI: 10.1107/S1600536810050178/bt5405Isup2.hkl

Additional supplementary materials:  crystallographic information; 3D view; checkCIF report

## Figures and Tables

**Table 1:** Hydrogen-bond geometry (Å, °)

*D*—H⋯*A*	*D*—H	H⋯*A*	*D*⋯*A*	*D*—H⋯*A*
O1—H1⋯N5^i^	0.82	1.93	2.743 (3)	172
N3—H3*A*⋯N8^ii^	0.86	1.96	2.807 (3)	166

Symmetry codes: (i) 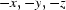; (ii) 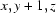.

**Fig. 1.**
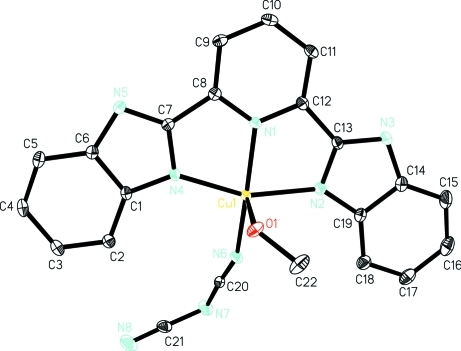
The molecular structure of the title compound, with atom labels and 30% probability displacement ellipsoids. All H atoms have been omitted.

**Fig. 2.**
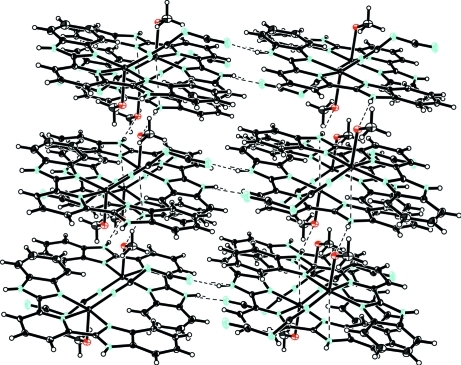
The packing diagram of the title compound.

## supplementary crystallographic information

### Comment

Benzimidazole derivatives and their metal complexes have attracted considerable interest over several decades, because many of these materials have been applied to various fields such as biological systems (Khaled, 2003; Hay *et al.*, 1998), Luminescent (Petoud *et al.*, 1997; Liu *et al.*, 2005), and magnetic properties (Boinnard *et al.*, 1990; Mo *et al.*, 2009). 2,6-bis(2-benzimidazol-2-yl)pyridine(bbp) (Addison *et al.*, 1981) as a benzimidazole derivative, is a tridentate ligand with two benzimidazole and one pyridine nitrogen atoms. In this paper, we report the structue of a new Cu^II^ complex based on bbp ligand.

Figure 1 shows that the Cu^II^ atom is coordinated by  N1, N2 and N4 from the tridentate bbp ligand and N6 from the dicyanamide anion, and O1 from one methanol molecule.  The distance of Cu-N bonds range from 1.9528 (19) to 2.0364 (2) Å. While the Cu-N1, Cu-N2 and Cu-N4 distances are 1.9763 (18), 2.0364 (19), 1.9955 (19) Å, respectively; and the N1-Cu-N2 and N1-Cu-N4 angles are 79.36 (7), 79.51 (7)°, respectively, these parameters are similar to those reported for other bbp-containing complexes (Wang *et al.*, 1994; Bernardinelli *et al.*, 1990). Due to intermolecular hydrogen-bonding interactions, the crystal structure is extended to a three-dimensional network (Figure 2).

### Experimental

The bbp (0.1 mmol) and CuClO_4_.6H_2_O (0.1 mmol) were added to 3 ml dimethylformamide with thorough stirring for 2 minutes. After filtering, the filtrate was carefully layered with 0.5 ml dimethylformamide and 5 ml methanol solution of Sodium dicyanamide (0.2 mol L^-1^), in turn. Green crystals suitable for X-ray analysis were obtained after one week. Elemental analysis found: C 55.56, H 3.33, N 23.46%; calculated for C_22_H_16_CuN_8_O: C 55.98, H 3.42, N 23.75%.

### Refinement

H atoms were positioned geometrically with C-H(phenyl, pyridyl) = 0.93 Å or 0.96 Å (methyl) and N-H = 0.8601 Å and refined using a riding model, with *U*_iso_(H) = 1.2*U*_eq_(C)_phenyl_, _pyridyl_, 1.2*U*_eq_(N)  or 1.5*U*_eq_(C)_methyl_.

### Crystal data

**Table:**

[Cu(C_19_H_12_N_5_)(C_2_N_3_)(CH_4_O)]	*Z* = 2
*M**_r_* = 471.98	*F*(000) = 482
Triclinic, *P*1	*D*_x_ = 1.566 Mg m^−^^3^
*a* = 6.8262 (14) Å	Mo *K*α radiation, λ = 0.71073 Å
*b* = 12.189 (2) Å	Cell parameters from 4368 reflections
*c* = 12.609 (3) Å	θ = 3.5–29.1°
α = 101.74 (3)°	µ = 1.13 mm^−^^1^
β = 99.03 (3)°	*T* = 293 K
γ = 97.12 (3)°	Block, green
*V* = 1001.2 (4) Å^3^	0.2 × 0.16 × 0.12 mm

### Data collection

**Table:**

Rigaku Saturn724 diffractometer	3591 independent reflections
Radiation source: fine-focus sealed tube	3294 reflections with *I* > 2σ(*I*)
graphite	*R*_int_ = 0.020
ω scans	θ_max_ = 25.3°, θ_min_ = 3.4°
Absorption correction: multi-scan (*CrystalClear*; Rigaku, 2007)	*h* = −8→7
*T*_min_ = 0.806, *T*_max_ = 0.874	*k* = −14→14
7787 measured reflections	*l* = −15→13

### Refinement

**Table:**

Refinement on *F*^2^	Primary atom site location: structure-invariant direct methods
Least-squares matrix: full	Secondary atom site location: difference Fourier map
*R*[*F*^2^ > 2σ(*F*^2^)] = 0.030	Hydrogen site location: inferred from neighbouring sites
*wR*(*F*^2^) = 0.072	H-atom parameters constrained
*S* = 1.02	*w* = 1/[σ^2^(*F*_o_^2^) + (0.0326*P*)^2^ + 0.7506*P*] where *P* = (*F*_o_^2^ + 2*F*_c_^2^)/3
3591 reflections	(Δ/σ)_max_ < 0.001
290 parameters	Δρ_max_ = 0.52 e Å^−^^3^
0 restraints	Δρ_min_ = −0.28 e Å^−^^3^

### Special details

**Table:**

Geometry. All esds (except the esd in the dihedral angle between two l.s. planes) are estimated using the full covariance matrix. The cell esds are taken into account individually in the estimation of esds in distances, angles and torsion angles; correlations between esds in cell parameters are only used when they are defined by crystal symmetry. An approximate (isotropic) treatment of cell esds is used for estimating esds involving l.s. planes.
Refinement. Refinement of F^2^ against ALL reflections. The weighted R-factor wR and goodness of fit S are based on F^2^, conventional R-factors R are based on F, with F set to zero for negative F^2^. The threshold expression of F^2^ > 2sigma(F^2^) is used only for calculating R-factors(gt) etc. and is not relevant to the choice of reflections for refinement. R-factors based on F^2^ are statistically about twice as large as those based on F, and R- factors based on ALL data will be even larger.

### Fractional atomic coordinates and isotropic or equivalent isotropic displacement parameters (Å^2^)

**Table:**

	*x*	*y*	*z*	*U*_iso_*/*U*_eq_	
Cu1	0.40402 (4)	0.04545 (2)	0.20901 (2)	0.01736 (10)	
O1	0.1127 (2)	0.03746 (13)	0.27080 (12)	0.0254 (4)	
H1	0.0241	0.0546	0.2282	0.030*	
N1	0.3511 (3)	0.14654 (14)	0.10718 (14)	0.0168 (4)	
N2	0.5225 (3)	0.19964 (14)	0.31068 (14)	0.0196 (4)	
N3	0.5742 (3)	0.38708 (14)	0.32891 (15)	0.0225 (4)	
H3A	0.5719	0.4521	0.3125	0.027*	
N4	0.2876 (3)	−0.06889 (14)	0.06878 (14)	0.0178 (4)	
N5	0.1565 (3)	−0.09661 (14)	−0.11513 (14)	0.0188 (4)	
N6	0.5284 (3)	−0.05076 (15)	0.29717 (15)	0.0246 (4)	
N7	0.7320 (3)	−0.19234 (16)	0.34181 (17)	0.0307 (5)	
N8	0.6356 (4)	−0.39938 (18)	0.2773 (2)	0.0430 (6)	
C1	0.2400 (3)	−0.18446 (17)	0.02561 (17)	0.0171 (4)	
C2	0.2565 (3)	−0.27628 (17)	0.07551 (19)	0.0203 (5)	
H2A	0.3067	−0.2652	0.1505	0.024*	
C3	0.1956 (3)	−0.38377 (18)	0.0091 (2)	0.0247 (5)	
H3B	0.2048	−0.4464	0.0401	0.030*	
C4	0.1200 (3)	−0.40094 (19)	−0.1044 (2)	0.0260 (5)	
H4A	0.0827	−0.4747	−0.1469	0.031*	
C5	0.0996 (3)	−0.31132 (19)	−0.15430 (19)	0.0238 (5)	
H5B	0.0478	−0.3233	−0.2292	0.029*	
C6	0.1599 (3)	−0.20125 (17)	−0.08783 (17)	0.0181 (4)	
C7	0.2323 (3)	−0.02371 (17)	−0.01919 (17)	0.0166 (4)	
C8	0.2646 (3)	0.10090 (17)	0.00213 (17)	0.0172 (4)	
C9	0.2181 (3)	0.17004 (18)	−0.07045 (18)	0.0203 (5)	
H9A	0.1545	0.1389	−0.1431	0.024*	
C10	0.2695 (3)	0.28666 (18)	−0.03130 (19)	0.0225 (5)	
H10A	0.2409	0.3345	−0.0785	0.027*	
C11	0.3629 (3)	0.33284 (18)	0.07713 (19)	0.0211 (5)	
H11A	0.3985	0.4110	0.1033	0.025*	
C12	0.4016 (3)	0.25952 (17)	0.14528 (18)	0.0185 (5)	
C13	0.4982 (3)	0.28491 (17)	0.26130 (18)	0.0186 (5)	
C14	0.6564 (3)	0.36794 (18)	0.42919 (19)	0.0240 (5)	
C15	0.7569 (4)	0.4417 (2)	0.5265 (2)	0.0351 (6)	
H15A	0.7786	0.5200	0.5337	0.042*	
C16	0.8230 (4)	0.3930 (2)	0.6122 (2)	0.0400 (7)	
H16A	0.8917	0.4398	0.6788	0.048*	
C17	0.7900 (4)	0.2751 (2)	0.6023 (2)	0.0343 (6)	
H17A	0.8374	0.2457	0.6621	0.041*	
C18	0.6886 (4)	0.2019 (2)	0.50539 (19)	0.0272 (5)	
H18A	0.6655	0.1238	0.4991	0.033*	
C19	0.6224 (3)	0.24939 (18)	0.41759 (18)	0.0209 (5)	
C20	0.6166 (3)	−0.12164 (18)	0.31518 (17)	0.0205 (5)	
C21	0.6717 (4)	−0.3023 (2)	0.3061 (2)	0.0273 (5)	
C22	0.1031 (4)	0.0833 (2)	0.3824 (2)	0.0383 (6)	
H22A	0.1013	0.1634	0.3931	0.057*	
H22B	0.2185	0.0705	0.4297	0.057*	
H22C	−0.0169	0.0470	0.3999	0.057*	

### Atomic displacement parameters (Å^2^)

**Table:**

	*U*^11^	*U*^22^	*U*^33^	*U*^12^	*U*^13^	*U*^23^
Cu1	0.02205 (16)	0.01322 (14)	0.01587 (15)	0.00307 (10)	0.00051 (11)	0.00332 (10)
O1	0.0220 (8)	0.0372 (9)	0.0165 (8)	0.0072 (7)	0.0009 (7)	0.0057 (7)
N1	0.0174 (9)	0.0157 (9)	0.0182 (9)	0.0033 (7)	0.0042 (7)	0.0049 (7)
N2	0.0220 (10)	0.0169 (9)	0.0190 (10)	0.0037 (7)	0.0017 (8)	0.0033 (7)
N3	0.0276 (10)	0.0132 (9)	0.0256 (11)	0.0029 (8)	0.0044 (8)	0.0026 (7)
N4	0.0181 (9)	0.0161 (9)	0.0180 (9)	0.0017 (7)	0.0021 (8)	0.0031 (7)
N5	0.0165 (9)	0.0222 (9)	0.0171 (9)	0.0033 (7)	0.0029 (8)	0.0029 (7)
N6	0.0330 (11)	0.0158 (9)	0.0234 (10)	0.0036 (8)	−0.0005 (9)	0.0055 (8)
N7	0.0317 (12)	0.0227 (10)	0.0371 (12)	0.0092 (9)	−0.0029 (9)	0.0101 (9)
N8	0.0559 (16)	0.0243 (12)	0.0550 (16)	0.0137 (11)	0.0164 (13)	0.0145 (10)
C1	0.0143 (10)	0.0155 (10)	0.0203 (11)	0.0013 (8)	0.0048 (9)	0.0007 (8)
C2	0.0178 (11)	0.0203 (11)	0.0225 (12)	0.0026 (9)	0.0031 (9)	0.0045 (9)
C3	0.0220 (12)	0.0171 (11)	0.0344 (14)	0.0023 (9)	0.0060 (10)	0.0047 (9)
C4	0.0228 (12)	0.0175 (11)	0.0318 (13)	−0.0002 (9)	0.0029 (10)	−0.0037 (9)
C5	0.0195 (12)	0.0258 (12)	0.0214 (12)	0.0005 (9)	0.0028 (10)	−0.0027 (9)
C6	0.0145 (11)	0.0201 (11)	0.0194 (11)	0.0019 (8)	0.0057 (9)	0.0026 (8)
C7	0.0142 (10)	0.0195 (10)	0.0166 (11)	0.0039 (8)	0.0030 (9)	0.0044 (8)
C8	0.0133 (11)	0.0200 (11)	0.0186 (11)	0.0021 (8)	0.0048 (9)	0.0040 (8)
C9	0.0188 (11)	0.0266 (12)	0.0176 (11)	0.0048 (9)	0.0047 (9)	0.0080 (9)
C10	0.0218 (12)	0.0249 (12)	0.0272 (12)	0.0087 (9)	0.0083 (10)	0.0145 (9)
C11	0.0211 (12)	0.0170 (11)	0.0281 (12)	0.0063 (9)	0.0074 (10)	0.0079 (9)
C12	0.0170 (11)	0.0172 (10)	0.0222 (12)	0.0041 (9)	0.0061 (9)	0.0040 (8)
C13	0.0203 (11)	0.0143 (10)	0.0217 (11)	0.0032 (8)	0.0066 (9)	0.0029 (8)
C14	0.0231 (12)	0.0228 (11)	0.0241 (12)	0.0029 (9)	0.0052 (10)	0.0006 (9)
C15	0.0378 (15)	0.0275 (13)	0.0315 (14)	−0.0026 (11)	0.0034 (12)	−0.0057 (10)
C16	0.0400 (16)	0.0421 (15)	0.0256 (14)	−0.0023 (13)	−0.0029 (12)	−0.0080 (11)
C17	0.0317 (14)	0.0474 (16)	0.0207 (13)	0.0069 (12)	−0.0010 (11)	0.0051 (11)
C18	0.0277 (13)	0.0302 (13)	0.0228 (13)	0.0058 (10)	0.0022 (10)	0.0053 (10)
C19	0.0188 (11)	0.0222 (11)	0.0195 (12)	0.0026 (9)	0.0028 (9)	0.0009 (9)
C20	0.0255 (12)	0.0174 (11)	0.0157 (11)	−0.0016 (9)	0.0006 (9)	0.0023 (8)
C21	0.0322 (14)	0.0256 (13)	0.0295 (13)	0.0108 (10)	0.0080 (11)	0.0128 (10)
C22	0.0356 (15)	0.0572 (17)	0.0196 (13)	0.0053 (13)	0.0073 (11)	0.0028 (12)

### Geometric parameters (Å, °)

**Table:**

Cu1—N6	1.9528 (19)	C3—H3B	0.9300
Cu1—N1	1.9763 (18)	C4—C5	1.378 (3)
Cu1—N4	1.9955 (19)	C4—H4A	0.9300
Cu1—N2	2.0364 (19)	C5—C6	1.403 (3)
Cu1—O1	2.2452 (16)	C5—H5B	0.9300
O1—C22	1.420 (3)	C7—C8	1.470 (3)
O1—H1	0.8200	C8—C9	1.392 (3)
N1—C8	1.336 (3)	C9—C10	1.387 (3)
N1—C12	1.344 (3)	C9—H9A	0.9300
N2—C13	1.330 (3)	C10—C11	1.384 (3)
N2—C19	1.388 (3)	C10—H10A	0.9300
N3—C13	1.348 (3)	C11—C12	1.381 (3)
N3—C14	1.378 (3)	C11—H11A	0.9300
N3—H3A	0.8601	C12—C13	1.461 (3)
N4—C7	1.358 (3)	C14—C15	1.386 (3)
N4—C1	1.380 (3)	C14—C19	1.408 (3)
N5—C7	1.331 (3)	C15—C16	1.377 (4)
N5—C6	1.389 (3)	C15—H15A	0.9300
N6—C20	1.151 (3)	C16—C17	1.404 (4)
N7—C20	1.296 (3)	C16—H16A	0.9300
N7—C21	1.315 (3)	C17—C18	1.380 (3)
N8—C21	1.148 (3)	C17—H17A	0.9300
C1—C2	1.399 (3)	C18—C19	1.390 (3)
C1—C6	1.412 (3)	C18—H18A	0.9300
C2—C3	1.378 (3)	C22—H22A	0.9600
C2—H2A	0.9300	C22—H22B	0.9600
C3—C4	1.406 (3)	C22—H22C	0.9600
			
N6—Cu1—N1	164.13 (8)	N5—C7—C8	127.12 (19)
N6—Cu1—N4	100.45 (8)	N4—C7—C8	116.10 (18)
N1—Cu1—N4	79.51 (7)	N1—C8—C9	120.47 (19)
N6—Cu1—N2	98.73 (8)	N1—C8—C7	110.70 (18)
N1—Cu1—N2	79.36 (7)	C9—C8—C7	128.83 (19)
N4—Cu1—N2	158.46 (7)	C10—C9—C8	118.1 (2)
N6—Cu1—O1	97.03 (7)	C10—C9—H9A	121.0
N1—Cu1—O1	98.82 (7)	C8—C9—H9A	121.0
N4—Cu1—O1	93.63 (7)	C11—C10—C9	120.9 (2)
N2—Cu1—O1	93.64 (7)	C11—C10—H10A	119.6
C22—O1—Cu1	122.14 (14)	C9—C10—H10A	119.6
C22—O1—H1	111.5	C12—C11—C10	118.1 (2)
Cu1—O1—H1	111.4	C12—C11—H11A	121.0
C8—N1—C12	121.54 (18)	C10—C11—H11A	121.0
C8—N1—Cu1	119.15 (14)	N1—C12—C11	120.9 (2)
C12—N1—Cu1	119.31 (14)	N1—C12—C13	109.69 (18)
C13—N2—C19	105.85 (17)	C11—C12—C13	129.39 (19)
C13—N2—Cu1	112.46 (14)	N2—C13—N3	112.53 (19)
C19—N2—Cu1	141.69 (15)	N2—C13—C12	119.10 (18)
C13—N3—C14	107.20 (18)	N3—C13—C12	128.36 (19)
C13—N3—H3A	126.4	N3—C14—C15	131.6 (2)
C14—N3—H3A	126.4	N3—C14—C19	106.14 (19)
C7—N4—C1	103.53 (17)	C15—C14—C19	122.3 (2)
C7—N4—Cu1	114.48 (13)	C16—C15—C14	116.4 (2)
C1—N4—Cu1	141.98 (15)	C16—C15—H15A	121.8
C7—N5—C6	102.69 (17)	C14—C15—H15A	121.8
C20—N6—Cu1	156.97 (18)	C15—C16—C17	122.1 (2)
C20—N7—C21	120.2 (2)	C15—C16—H16A	118.9
N4—C1—C2	131.1 (2)	C17—C16—H16A	118.9
N4—C1—C6	107.57 (18)	C18—C17—C16	121.3 (2)
C2—C1—C6	121.29 (19)	C18—C17—H17A	119.4
C3—C2—C1	117.4 (2)	C16—C17—H17A	119.4
C3—C2—H2A	121.3	C17—C18—C19	117.5 (2)
C1—C2—H2A	121.3	C17—C18—H18A	121.3
C2—C3—C4	121.5 (2)	C19—C18—H18A	121.3
C2—C3—H3B	119.2	N2—C19—C18	131.3 (2)
C4—C3—H3B	119.2	N2—C19—C14	108.28 (19)
C5—C4—C3	121.7 (2)	C18—C19—C14	120.5 (2)
C5—C4—H4A	119.1	N6—C20—N7	173.5 (2)
C3—C4—H4A	119.1	N8—C21—N7	174.3 (3)
C4—C5—C6	117.5 (2)	O1—C22—H22A	109.5
C4—C5—H5B	121.2	O1—C22—H22B	109.5
C6—C5—H5B	121.2	H22A—C22—H22B	109.5
N5—C6—C5	130.0 (2)	O1—C22—H22C	109.5
N5—C6—C1	109.42 (18)	H22A—C22—H22C	109.5
C5—C6—C1	120.6 (2)	H22B—C22—H22C	109.5
N5—C7—N4	116.78 (18)		

### Hydrogen-bond geometry (Å, °)

**Table:**

*D*—H···*A*	*D*—H	H···*A*	*D*···*A*	*D*—H···*A*
O1—H1···N5^i^	0.82	1.93	2.743 (3)	172.
N3—H3A···N8^ii^	0.86	1.96	2.807 (3)	166.

Symmetry codes: (i) −*x*, −*y*, −*z*; (ii) *x*, *y*+1, *z*.

## References

[bb1] Addison, A. W. & Burke, P. J. (1981). *J. Heterocycl. Chem.* **18**, 803–805.

[bb2] Bernardinelli, G., Hopfgartner, G. & Williams, A. F. (1990). *Acta Cryst.* C**46**, 1642–1645.

[bb3] Boinnard, D., Cassoux, P., Petrouleas, V., Savariault, J. M. & Tuchagues, J. P. (1990). *Inorg. Chem.* **29**, 4114–4122.

[bb4] Hay, R. W., Clifford, T. & Lightfoot, P. (1998). *Polyhedron*, **17**, 3575–3581.

[bb5] Khaled, K. F. (2003). *Electrochim. Acta*, **48**, 2493–2503.

[bb6] Liu, S. G., Zuo, J. L., Wang, Y., Li, Y. Z. & You, X. Z. (2005). *J. Phys. Chem. Solids*, **66**, 735–740.

[bb7] Mo, H. J., Zhong, Y. R., Cao, M. L., Ou, Y. C. & Ye, B. H. (2009). *Cryst. Growth. Des.* **9**, 488–496.

[bb8] Petoud, S., Bünzli, J. C. G., Schenk, K. J. & Piguet, C. (1997). *Inorg. Chem.* **36**, 1345–1353. 10.1021/ic961305a 11669711

[bb9] Rigaku (2007). *CrystalClear* Rigaku Corporation, Tokyo, Japan.

[bb10] Sheldrick, G. M. (2008). *Acta Cryst.* A**64**, 112–122. 10.1107/S0108767307043930 18156677

[bb11] Wang, S. X., Yu, S. Y., Luo, Q. H., Wang, Q. Y., Shi, J. Q. & Wu, Q. J. (1994). *Transition Met. Chem.* **19**, 205–208.

